# Linear erythematous papulovesicular rash in a newborn

**DOI:** 10.1016/j.jdcr.2025.01.019

**Published:** 2025-02-07

**Authors:** Jane Hong, Sherman Chu, Mary Cowden, Bethany R. Rohr, Timmie R. Sharma

**Affiliations:** a Case Western Reserve University School of Medicine, Cleveland, Ohio; b Department of Dermatology, University Hospitals Cleveland Medical Center, Cleveland, Ohio; c Department of Dermatology, MetroHealth Medical Center, Cleveland, Ohio

**Keywords:** Blaschkoid, ILVEN, inflammatory linear verrucous epidermal nevus

A 35 6 or 7-week diamniotic dichorionic twin admitted to the neonatal intensive care unit for respiratory distress presented at day 2 of life with an erythematous papulovesicular rash involving her right leg. On days 3 to 4, the rash spread to the right thigh, foot, and toes, in a linear pattern following the lines of Blaschko with overlying scale and erosions (Fig 1, Fig 2, Fig 3). Her fraternal twin brother presented with no similar findings and there was no family history of any similar rash. A viral swab was performed from vesicular fluid and negative for herpes simplex virus (HSV) by polymerase chain reaction. A punch biopsy was taken. There were no other systemic abnormalities on presentation.

**Fig 1:**
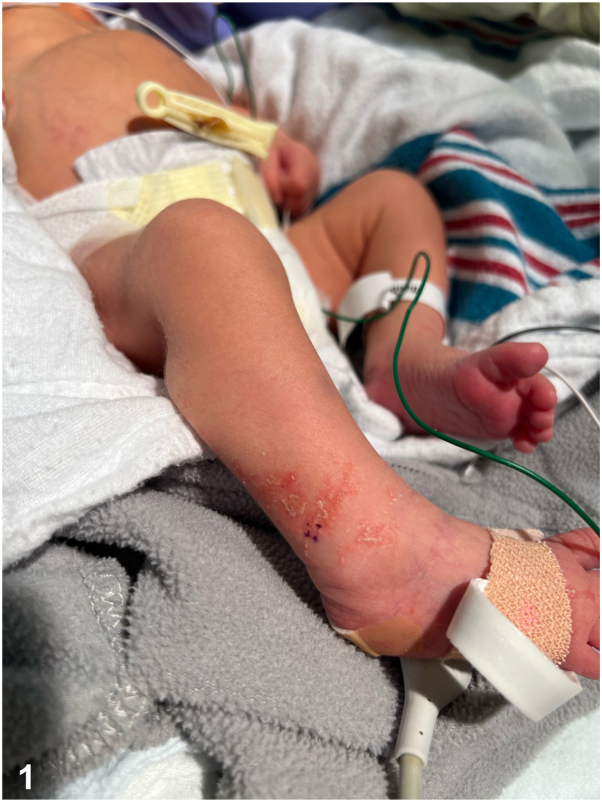

**Fig 2:**
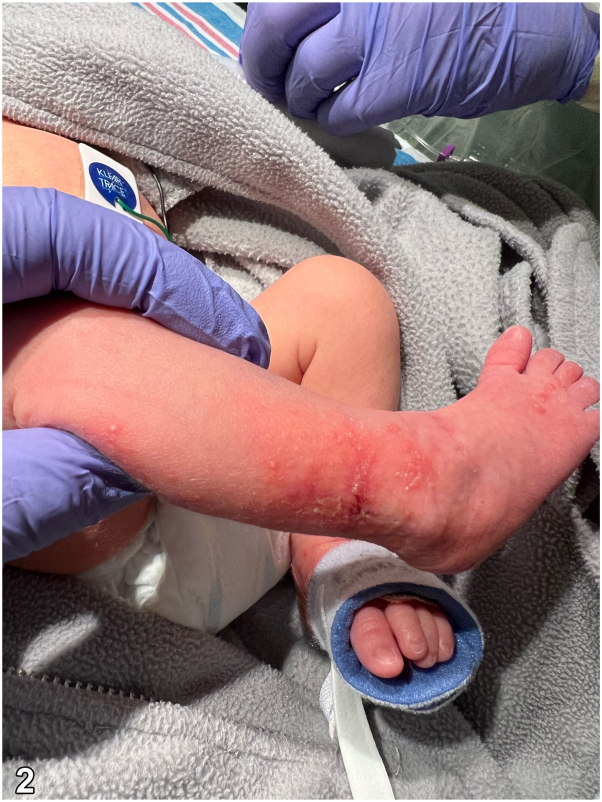

**Fig 3:**
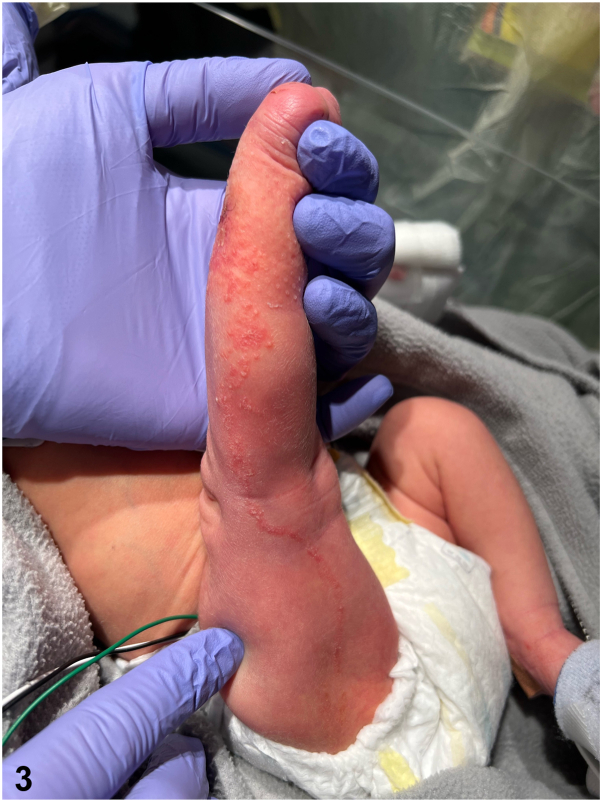

**Question 1: Which of the following is the most likely diagnosis?**

A. Incontinentia pigmenti
B. HSV
C. Inflammatory linear verrucous epidermal nevus (ILVEN)
D. Lichen striatus
E. Linear porokeratosis

**Answers:**

A. Incontinentia pigmenti – Incorrect. Incontinentia pigmenti, is a congenital X-linked dominant condition, which is characterized by 4 stages. The first vesiculobullous stage, lasting days to weeks, presents erythematous vesicles in a linear distribution on torso/extremities. Although some neonatal cases note neurological or ophthalmological abnormalities, this patient had no systemic findings such as seizures or family history of rash.^1^
B. HSV – Incorrect. HSV typically presents as grouped erythematous vesicles, not linear lesions in a Blaschkoid pattern. Additionally, the HSV polymerase chain reaction was negative, making this an unlikely diagnosis.
C. ILVEN – Correct. ILVEN is a rare variant of an epidermal nevus is typically seen in early childhood, but can occasionally present at birth. Lesions are typically pruritic, erythematous, verrucous papules, and plaques in a linear distribution following the lines of Blaschko, but can present as vesicles at early presentation.
D. Lichen striatus – Incorrect. Lichen striatus presents as flat-topped flesh-colored or hypopigmented papules along the lines of Blaschko. Lesions typically do not present at birth, but typically presents between 4 months and 15 years of life.^2^
E. Linear porokeratosis – Incorrect. Linear porokeratosis commonly appears on the extremities as unilateral hyperkeratotic papules in a linear pattern along the lines of Blaschko. Lesions usually present at early childhood.

**Question 2: What of the following histopathologic findings is seen with this diagnosis?**

A. Epidermal acanthosis with alternating orthokeratosis and parakeratosis
B. Eosinophilic spongiosis with dyskeratotic keratinocytes
C. Lichenoid dermatitis with involvement of sweat glands and hair follicles
D. Ballooning degeneration of keratinocytes and multinucleated giant cells
E. Cornoid lamella

**Answers:**

A. Epidermal acanthosis with alternating orthokeratosis and parakeratosis – Correct. This is a characteristic histopathologic feature of ILVEN.^1^
B. Eosinophilic spongiosis with dyskeratotic keratinocytes – Incorrect. This histology finding can be seen during the vesicular stage of incontinentia pigmenti.
C. Lichenoid dermatitis with involvement of sweat glands and hair follicles – Incorrect. This is the histologic presentation of lichen striatus.
D. Ballooning degeneration of keratinocytes and multinucleated giant cells – Incorrect. This finding is consistent with HSV infection.
E. Cornoid lamella - Incorrect. Cornoid lamella is a distinctive histologic feature for the diagnosis of all variants of porokeratoses.

**Question 3: Which of the following statements about this condition is true?**

A. Topical steroids, calcineurin inhibitors, and laser therapy are potential treatment options
B. There are 4 classic stages of evolution of this condition
C. This condition responds well to antiviral therapies
D. Squamous cell carcinoma can commonly arise from this condition
E. There is no known gene mutation associated with this condition

**Answers:**

A. Topical steroids, calcineurin inhibitors, and laser therapy are potential treatment options – Correct. There are multiple pharmacological and surgical modalities that benefit ILVEN symptoms to varying degrees.^3^
B. There are 4 classic stages of evolution of this condition – Incorrect. A 4 stage progression is associated with incontinentia pigmenti rather than ILVEN.
C. This condition responds well to antiviral therapies – Incorrect. Antivirals have not been shown to be beneficial in treating ILVEN.
D. Squamous cell carcinoma can commonly arise from this condition – Incorrect. Although it has a chronic course, squamous cell carcinoma is not associated with ILVEN.
E. There is no known gene mutation associated with this condition – Incorrect. ILVEN is associated with a CARD14 mutation.^1^

## Conflicts of interest

None disclosed.
